# Nurses’ strategies for coping with stress in the face of the influx of war refugees from Ukraine to Poland

**DOI:** 10.3389/fpsyt.2024.1479280

**Published:** 2024-10-22

**Authors:** Krystyna Kowalczuk, Katarzyna Tomaszewska, Andriej Szpakow, Elżbieta Krajewska-Kułak, Marek Sobolewski, Justyna Magdalena Hermanowicz

**Affiliations:** ^1^ Department of Integrated Medical Care, Medical University of Bialystok, Bialystok, Poland; ^2^ Department of Nursing, Institute of Health Protection, The Bronislaw Markiewicz State Higher School of Technology and Economics, Jaroslaw, Poland; ^3^ Department of Health Care, Prof. Edward F. Szczepanik State Vocational College, Suwalki, Poland; ^4^ Faculty of Management, Rzeszow University of Technology, Rzeszow, Poland; ^5^ Department of Pharmacodynamics, Medical University of Bialystok, Bialystok, Poland

**Keywords:** nurse, stress, coping strategy, war refugees, burden, work fatigue, EU’s mass refugee directive, Ukrainian refugees

## Abstract

**Introduction:**

Russian military’s incursion into Ukraine sparked the largest refugee crisis in Europe since World War II. As Ukraine’s neighboring country, Poland became the primary destination for these refugees. Ukrainians staying in Poland under the EU’s Temporary Protection Directive receive humanitarian support similarly to asylum seekers, but the legal pathways, length of stay and integration processes differ significantly as the Directive provides for more immediate, collective protection without the complexity of individual asylum applications. The influx of war refugees generates extremely complex situations that health personnel, especially nurses, must face on a daily basis.

**Aim:**

Identify whether and to what extent the emergence of a large number of patients with war-related experiences constituted a source of stress for nurses, and how Polish nurses coped with this issue.

**Materials and method:**

A cross-sectional study was conducted in December 2022 in Białystok, Poland. It included 473 certified nurses working in hospitals affiliated with the District Chamber of Nurses in Białystok. Perceived Stress Scale (PSS-10) questionnaire and Mini-COPE inventory (Polish version of Carver’a BriefCOPE inventory were used in the study.

**Results:**

The average stress level among nurses was moderate, with nearly equal proportions of nurses experiencing low, medium, and high stress levels. Nurses who had contact with refugees in the last six months and/or helped them, did not present an increased level of stress - on the contrary, this level was lower. Nurses who helped refugees at work more often used a planning strategy in stressful situations, and less frequently reacted to stress using humour, religion, denial or venting.

**Conclusions:**

The greater workload and more frequent contacts between Polish nurses and Ukrainian war refugees were a factor increasing work fatigue, but, paradoxically, at the same time reducing the level of stress in a situation giving the feeling of a duty well fulfilled. Empowering nurses through greater autonomy and fostering supportive work environments, especially in crisis situations like the care of war refugees has a positive impact on coping with stress.

## 1 Introduction

In the face of global conflicts and humanitarian crises, the influx of war refugees poses a monumental challenge to societies around the world. Poland has joined many other countries experiencing this phenomenon, which poses new and difficult tasks for various social sectors, especially for the health service (1).

In the context of health care, the influx of war refugees generates extremely complex situations that health personnel, especially nurses, must face on a daily basis (2). War refugees often bring not only physical injuries and illnesses related to the conflict, but also deep-seated psychological trauma. In the face of these challenges, medical staff must provide not only the comprehensive health care, but also emotional and psychosocial support in difficult moments of adaptation to the new environment (3).

The challenges faced by medical staff are multidimensional. Nurses must cope not only with the medical implications of patients’ war experiences, but also with their unique psychosocial needs resulting from trauma. Additionally, medical staff find themselves in a situation where there is often a lack of resources, both human and financial, which further complicates the provision of comprehensive care (4).

In the context of these difficult conditions, scientific theories of stress become not only a tool for understanding the psychosocial processes accompanying the work of nurses, but are also a key starting point for developing coping strategies and support for medical staff.

The Transactional Model of Stress and Coping by Richard Lazarus and Susan Folkman allows to delve into the subjective assessment of the situation, which is crucial for working with war refugee patients (5). This theory emphasizes the role of the subjective experience of stress, in which the way how an individual interprets a given situation influences whether it will be classified as stressful. With regard to nursing, where everyday challenges related to the care of war refugees are complex and ambiguous, this model allows to understand what factors influence the subjective feeling of stress experienced by medical staff.

The Stress Process Model developed by Richard S. Schulz (6), often used in the context of caregiving, outlines the dynamic interplay between stressors, resources, and outcomes in the caregiving experience. In this model stressors are the challenges, demands, or stress-inducing factors that caregivers encounter in their caregiving role. Stressors can include the physical, emotional, and financial burdens associated with caregiving, as well as the deterioration of the care recipient’s health. Resources refer to the internal and external assets that caregivers have at their disposal to cope with stressors. Internal resources might include coping skills, resilience, and social support networks, while external resources could include financial assistance, access to healthcare services, and community support programs.

Coping processes involve the strategies and mechanisms caregivers use to manage stressors and maintain their well-being. These coping strategies can be active (e.g. planning, positive reframing, avoidant (e.g. self-distraction, denial, substance use) or support-seeking/emotion-focused (e.g. religion, venting, self-blame). Mediating factors are variables that influence the relationship between stressors, resources, and outcomes. These might include factors such as caregiver characteristics (e.g., age, gender, personality), care recipient characteristics (e.g., illness severity, behavior), and contextual factors (e.g., cultural norms, healthcare policies). Outcomes refer to the effects of caregiving on the caregiver’s physical health, mental health, quality of life, and overall well-being. These outcomes can be both positive (e.g., personal growth, strengthened relationships) and negative (e.g., caregiver burden, depression, physical ailments).

The Richard S. Schulz Stress Process Model emphasizes the dynamic nature of caregiving, recognizing that stressors, resources, and outcomes interact and evolve over time. It underscores the importance of understanding the complex interplay between various factors in shaping the caregiving experience and its impact on caregivers’ lives.

On February 24, 2022, the Russian military’s incursion into Ukraine sparked the largest refugee crisis in Europe since World War II. As Ukraine’s neighboring country, Poland became the primary destination for these refugees. Between February 24, 2022, and February 24, 2023, a staggering 10.056 million refugees, primarily women and children, crossed into Poland. By January 2023, almost 2 million found refuge in private homes across the country. Unlike previous European migration crises, no refugee camps were established. Instead, Poland implemented a public funding program to support those who took refugees into their homes. Over 24% of war refugees from Ukraine have remained in Poland under the Temporary Protection Directive (7).

All Ukrainians crossing the Polish border post-invasion were granted access to healthcare services, which included ambulatory care, hospital treatments, drug reimbursements, medical device supplies, preventive vaccinations, and rehabilitation. The National Health Fund (NHF) financed healthcare services for Ukrainian citizens. By June 2022, 2,230 hospitalizations were recorded for Ukrainian patients, with 1,494 children, 578 women, and 158 men among them. During the first three months of the conflict, 27,861 refugees, 58% of whom were children, received care in Polish healthcare facilities, predominantly as outpatients. In 2022, the total social and healthcare costs Poland incurred for accepting Ukrainian refugees amounted to approximately 8.4 billion euros (8).

The reception of Ukrainian war refugees is in line with the EU Temporary Protection Directive (9), and has key similarities to the reception of other refugees/asylum seekers, such as access to basic services (healthcare, education), protection from deportation, and the right to work. Both groups often rely on support from NGOs or local governments. However, there are also key differences. Asylum seekers undergo lengthy individual processes, while Ukrainians receive immediate temporary protection without the need for asylum applications. Asylum seekers may stay indefinitely if granted refugee status, whereas Ukrainians’ stay is limited to four years under the directive. Ukrainians have more immediate access to services like education and employment, while asylum seekers may face delays until their application is approved. The directive specifically applies to Ukrainians, ensuring a faster, more unified response compared to the diverse origins and individualized cases of asylum seekers.

The collision of nurses with a vast number of people in need of any form of assistance could have been a source of stress for the nurses. To ascertain whether this was indeed the case, we decided to conduct research among nurses using the PSS-10 questionnaire (10). The PSS-10 questionnaire is a valuable tool for researchers and clinicians to assess perceived stress levels in individuals and to identify those who may be at risk for stress-related health problems. The Polish version of the PSS-10 is well validated and widely used (11).

In the context of stress coping strategies adopted by Polish nurses taking care of Ukrainian war refugees, we decided to use the common and recognized “MiniCOPE” questionnaire (11), which is the Polish adaptation of the “BriefCOPE” questionnaire by Terrence Carver (12), which is a shortened version of the COPE (Coping Orientation to Problems Experienced) questionnaire by the same author (13). This tool was developed based on the Transactional Model of Stress and Coping by Richard Lazarus and Susan Folkman (14). It allows for quick and concise identification of various forms of coping with stressful situations. The MiniCOPE questionnaire identifies both active strategies, such as acceptance or positive reframing, and avoidant ones, for instance, avoidance or substance use. The analysis of MiniCOPE allows to understand how individuals adapt different coping strategies depending on the context of the stressor. MiniCOPE is a tool measuring strategies for coping with stress, adapted to the Polish culture, which makes it more time-effective and practical in research conducted in the Polish population. The questionnaire helps to identify what coping strategies individuals prefer when facing stress, which is a valuable source of information, especially in the context of health care.

The aim of our research was primarily to identify whether and to what extent the emergence of a large number of patients with war-related experiences constituted a source of stress for nurses, and how Polish nurses coped with this issue. Such a study is as unique as the unprecedented influx of a huge number of war refugees in a relatively short period of time, which has never been seen before in Poland’s history. The results of our study may serve as a basis for designing support programs for medical personnel working with war refugees.

## 2 Materials and methods

A cross-sectional study was conducted in December 2022 in Białystok, Poland. It included 473 certified nurses working in hospitals affiliated with the District Chamber of Nurses in Białystok.

### 2.1 Ethical considerations

The ethical challenges in this study stem from issues related to participant consent, confidentiality, potential psychological distress, and the voluntary nature of participation. To minimize ethical risks all participants were informed about the nature, purpose, and procedures of the study. Their participation was voluntary, and they had the right to withdraw at any time without consequences. The participants were ensured that their responses will be kept anonymous and confidential, with no identifying information linked to the data collected.

The study received approval from the Bioethics Committee of the Medical University of Białystok, acting in accordance with the Declaration of Helsinki, ref. no. APK.002.434.2022, ensuring that all procedures followed ethical standards for research involving human subjects.

The authors considered the benefits to outweigh the risks, as the findings provide valuable insights into improving nurse support systems, stress management strategies, and healthcare policies for vulnerable populations. The potential stress from participating was mitigated by the significant contribution the research makes to better healthcare practices and support for nurses working in crisis situations.

### 2.2 Selection of the study group and study protocol

The selection criterion was full-time work in hospital based on a contract of employment. To ensure consistency in workload, exposure, and institutional support, minimizing variability and confounding factors that could affect the study*’*s reliability and comparability nurses working part-time and on a basis other than a contract of employment were excluded from the study.

The study was conducted using paper questionnaires. The surveys were distributed to nurses by researchers during training organized by the District Chamber of Nurses in Białystok. Nurses were not asked about their knowledge of Ukrainian or Russian. In the north-eastern region of Poland, where the study was conducted, the vast majority of residents, including nurses, can communicate freely with Ukrainians. The nurses were asked to complete the surveys in their free time and to return the surveys in a closed, sealed envelope. 600 questionnaires were distributed, 473 correctly completed questionnaires were returned, which gave a response rate of 78.8*%*. The reasons why 127 respondents withdrew from the study are unknown. A margin of error of +/- 5% was assumed at a confidence level of 0.95, which gives a minimum sample size of 384. The number of surveys collected exceeds this value, making the estimation of the frequency of contact with refugees even more precise. All demographic and professional data were obtained from self-report questionnaires. No incentives were used to encourage nurses to participate in the study.

### 2.3 Description of questionnaires and measures

The ability to cope with stress was measured using the research tool - multidimensional Mini-COPE inventory (11), which is the Polish version of BriefCOPE: a shortened version of Carver*’*s COPE inventory (13). Carver designed her multidimensional COPE inventory based on the Lazarus stress model (14) and the behavioural self-regulation model by Scheier and Carver (13). The Mini-COPE inventory measures 14 coping facets. They can be grouped into three key coping strategies: active coping, avoidant coping, and support-seeking/emotion-focused coping. Active strategies include the following facets: active coping, planning and positive reframing. Avoidant strategies include acceptance, humour, self-distraction, denial, substance use, and behavioural disengagement. The support-seeking/emotion-focused strategy includes religion, emotional support, instrumental support, venting and self-blame. Each answer can be rated on a scale from 0 (not used at all) to 3 (often used). The measures were standardized by the authors of the questionnaire. The original BriefCOPE inventory and its Polish version Mini-COPE have been extensively validated and have clear scoring guidelines.

The second questionnaire used in the study was the internationally recognized Perceived Stress Scale (PSS-10), validated for the Polish language and conditions. PSS-10 allows to assess subjective feelings and thoughts related to the problem. The scale consists of 10 questions answered on a 5-point scale starting from 0 (never) to 4 (very often). In the case of four positive answers, the score between 20-40 indicates a high level of stress, 14-19 - a moderate level, and 0-13 - a low level of stress.

### 2.4 Statistical methods

The chi-square test of independence was used to assess the significance of differences. To compare the significance of differences in the use of coping strategies (Mini-COPE measures) in both groups, the Mann-Whitney test was applied for detailed measurements, and the t-test was used for groups of strategies (due to closeness - normal distributions). Normality of the distribution was tested using the Shapiro-Wilk test, as well as viewing normal probability plots. Attention was also paid to the coefficient of skewness. In addition to the parametric t test, the results were also checked using a nonparametric test. In a similar way, differences in Mini-COPE measures were assessed depending on the contact with refugees.

Regression analysis was used to thoroughly examine the relationship between the elements related to the contact with refugees and the use of strategies for coping with stress, including control factors, such as nurses’ age, education and marital status. Stepwise regression was applied to select optimal models that included only statistically significant variables.

The STATISTICA v.13 software was used for calculations. To facilitate the interpretation of the results, statistically significant results were marked according to the following convention: p < 0.05 (*), p < 0.01 (**), p < 0.001 (***).

## 3 Results

### 3.1 Study group

In the survey participated 473 nurses. The vast majority of the respondents were women - 85%. The average age of the respondents was 41 years of age, with over half of them being over 42 years old. Almost two thirds of the nurses were married. More than half of the respondents had children. Almost every second nurse had a master’s degree, every third – a bachelor’s degree and every fifth - secondary vocational education.

Among the surveyed medical staff, every third person had contact with war refugees. Almost 30% of the respondents admitted that their medical facility provided assistance to war refugees. Every tenth person took up additional work due to the influx of war refugees to Poland.

### 3.2 The level of stress

The level of nurses’ stress was assessed using the PSS-10 questionnaire. The **Table 1** shows the distribution of the PSS-10 numerical measures and **Table 2** shows the classification of nurses’ well-being into three groups. In total 467 nurses completed the PSS-10 questionnaire and 6 nurses did not complete the questionnaire (**Table 2**).

**Table 1:** Distribution of the PSS-10 numerical measures.

PSS-10						
Mean	Median	Std. dev.	Low quartile	Upper quartile	Min	Max
16.6	17	6.1	12	21	0	34

**Table 2:** Classification of nurses’ well-being into three groups.

Stress level	Number	Percent
low	146	31.3%
medium	169	36.2%
high	152	32.5%

### 3.3 The level of stress and selected factors

The correlation of the level of stress determined using the PSS-10 numerical measure and contact with war refugees at work has been examined. The intensity of contact with refugees was categorized as follows: contact with war refugees in the last 6 months (direct contact with refugees either at work or outside of it), helping war refugees in the workplace (providing care to refugees), working in a medical facility where war refugees stayed (without providing direct care to them), and taking up additional work to help refugees (engaging in additional duties due to refugees). The **Table 3** below presents detailed descriptive statistics showing the distribution of the PSS-10 measures depending on the contact with refugees at work. It also presents other questions regarding relationships with war refugees.

**Table 3:** Descriptive statistics showing the distribution of the PSS-10 measures depending on the contact with refugees at work and in other contacts.

Information about contact with refugees	PSS-10						*p*
	No			Yes			
	*N*	Mean (95% c.i.)	Std. dev.	*N*	Mean (95% c.i.)	Std. dev.	
Contact with war refugees in the last 6 months	309	17.0 (16.3–17.6)	6.0	158	15.9 (14.9–16.8)	6.2	0.0660
Helping war refugees in the workplace	327	17.0 (16.3–17.6)	6.2	129	15.7 (14.7–16.7)	5.8	0.0446*
Work in a medical facility where war refugees stayed	314	16.7 (16.0–17.4)	6.1	149	16.3 (15.3–17.3)	6.1	0.4985
Taking up additional work to help refugees	410	16.6 (16.0–17.2)	6.1	48	16.3 (14.6–17.9)	5.8	0.7251

p – probability value calculated using the independent samples t-test. * p<0.05.

The relationship between contacts with refugees and the intensity of stress shown above may be the result of some other hidden factor - perhaps refugees are helped by younger people who may have a lower level of stress. Therefore, the general regression model was used, in which the following were entered as independent variables: age, education, having children, contact with refugees in the last 6 months or helping refugees at work. Various procedures (including stepwise regression) were applied in search for an optimal model, which would include the factor related to contacts with refugees and other statistically significant factors.

The two models were obtained, which confirm the results of the elemental univariate analysis presented in **Table 3**. Both regression models explain a very small part of the variability of stress levels in the studied population. Among the sociodemographic data collected in the survey, there were no features that would allow obtaining higher values of the coefficient of determination. However, the main goal of the regression analysis was to verify the results contained in **Table 3**. More precisely, to determine whether the differences observed between people in contact with refugees and the rest in terms of the PSS-10 are not apparent. Despite the fact that the coefficient of determination turned out to be very low in the regression models presented in **Tables 4** and **5**, it is sufficient for the models to confirm to a large extent the results presented in **Table 3**. The results of the models are presented in **Tables 4**, **5**.

**Table 4:** Stress level among nurses having contact with refugees in the last 6 months.

Independent factors	PSS-10 *R* ^2^ = 3.8% *F* = 6.2 *p* = 0.0004***		
	*B* (95% c.i.)	*p*	*ß*
age (years)	-0.071 (-0.116; -0.025)	0.0026**	-0.14
master’s degree vs. other form of education	-1.636 (-2.745; -0.526)	0.0040**	-0.13
contact with refugees in the last 6 months	-1.000 (-2.154; 0.155)	0.0896	-0.08

R ^2^ – coefficient of determination, F – statistical test and p value for significance of the entire model.

B – regression coefficient with 95% c.i., p value for significance of each regression coefficient.

ß – standardized regression coefficient. ** p<0.01.

**Table 5:** Stress level among nurses helping refugees in the workplace.

Independent factors	PSS-10 *R* ^2^ = 3.9% *F* = 6.1 *p* = 0.0004***		
	*B* (95% c.i.)	*p*	*ß*
age (years)	-0.071 (-0.118; -0.025)	0.0026**	-0.14
master’s degree vs. other form of education	-1.560 (-2.688; -0.433)	0.0068**	-0.13
helping refugees in the workplace	-1.075 (-2.309; 0.159)	0.0875	-0.08

R^2^ – coefficient of determination, F – statistical test and p value for significance of the entire model.

B – regression coefficient with 95% c.i., p value for significance of each regression coefficient.

ß – standardized regression coefficient.

First of all, in both models the factor related to the contact with or helping refugees was close to statistical significance (p below 0.10), and the value of the regression coefficient shows that people having contact with refugees (or in the **Table 5** - providing help to refugees) have by approximately 1 point lower level of stress. Additionally, the fact of having a master’s degree (decrease by approximately 1.6 point) and age (decrease by approximately 0.7 point for every 10 years) reduce the level of stress.

An interesting complement to the analysis presented in **Tables 4** and **5** may be showing the distribution of stress levels (low, medium, high) depending on the contact with war refugees. Such a summary was prepared for two forms of the contact with refugees, which were associated with significantly different levels of the PSS-10 measure. Results are presented in **Tables 6**, **7**.

**Table 6:** Distribution of stress levels depending on the contact with war refugees.

Stress level	Contact with war refugees in the last 6 months (*p* = 0.2085)	
	No	Yes
low	89 (28.8%)	57 (36.1%)
medium	119 (38.5%)	50 (31.6%)
high	101 (32.7%)	51 (32.3%)

**Table 7:** Distribution of stress levels depending on helping refugees in the workplace.

Stress level	Helping war refugees in the workplace (*p* = 0.0519)	
	No	Yes
low	91 (27.8%)	51 (39.5%)
medium	123 (37.6%)	41 (31.8%)
high	113 (34.6%)	37 (28.7%)

The significance of differences in stress levels between the groups was compared using the chi-square test of independence. The results shown in the **Table 7** are noteworthy, because the relationship is on the verge of statistical significance (p = 0.0519) and there is an over 10% greater share of people with a low level of stress among nurses helping refugees in the workplace.

The data from 145 nurses, who worked with refugees in a medical facility, was used to determine whether the level of stress was influenced by the declaration that working with refugees was a burden. The **Table 8** shows the characteristics of the PSS-10 distribution depending on the feeling of burden in the work with war refugees.

**Table 8:** Characteristics of the PSS-10 distribution depending on the feeling of burden in the work with war refugees.

Was working with refugees a burden?						*p*
No			Yes			
*N*	Mean (95% c.i.)	Std. dev.	*N*	Mean (95% c.i.)	Std. dev.	
97	16.9 (15.7–18.1)	5.7	48	15.0 (13.1–16.9)	6.6	0.0771

p – probability value calculated using the independent samples t-test.

### 3.4 Stress-management strategies according to Mini-COPE

The Mini-COPE questionnaire was applied to estimate the frequency of using certain measures of behaviour in stressful situations. The frequency of using each strategy was rated on a scale from 0 to 3 points. **Table 9** presents a summary of basic descriptive statistics applied to assess the frequency of using particular strategies in a stressful situation.

**Table 9:** Descriptive statistics of using by nurses particular strategies in a stressful situation.

Mini-COPE	Mean	Median	Std. dev.	Min	Max
Active coping	2.09	2	0.61	0.5	3
Planning	2.06	2	0.67	0	3
Positive reframing	1.77	2	0.70	0	3
Acceptance	1.87	2	0.66	0	3
Humor	0.92	1	0.69	0	3
Religion	1.28	1	0.97	0	3
Seeking emotional support	1.91	2	0.77	0	3
Seeking instrumental support	1.81	2	0.81	0	3
Self-distraction	1.78	2	0.68	0	3
Denial	0.90	1	0.69	0	3
Venting	1.32	1.5	0.65	0	3
Substance use	0.42	0	0.72	0	3
Behavioral disengagement	0.78	0.5	0.66	0	3
Self-blame	1.14	1	0.79	0	3

As presented, the frequency of using active, (constructive) strategies is much higher than avoidant (destructive) ones – denial, substance use, or support-seeking/emotion-focused (passive) strategies – self-blame or venting.

### 3.5 Correlations between Mini-COPE stress coping strategies and nurses’ contacts with refugees

The relationship between the use of stress-management strategies at work with refugees and the fact of providing help to refugees was examined. The non-parametric Mann-Whitney test was used for the analysis. The choice of the non-parametric test was dictated by the relatively large asymmetry of most Mini-COPE measures. For the same reason, the tables also include the median value, which may be a better measure of the average level than the arithmetic mean.

**Table 10**. shows that the people who helped refugees at work more often used a planning strategy in stressful situations, and less frequently reacted to stress using humour, religion, denial or venting. There were no statistically significant relationships for the remaining strategies.

**Table 10:** Coping strategies among nurses helping war refugees in the workplace.

Mini-COPE	Helping war refugees in the workplace						*p*
	no (*N* = 327)			yes (*N* = 131)			
	Median	Mean (95% c.i.)	Std. dev.	Median	Mean (95% c.i.)	Std. dev.	
Active coping	2.00	2.08 (2.02-2.15)	0.60	2.00	2.13 (2.02-2.23)	0.63	0.3959
Planning	2.00	1.99 (1.92-2.07)	0.67	2.00	2.24 (2.13-2.35)	0.62	0.0006***
Positive reframing	2.00	1.74 (1.67-1.82)	0.69	2.00	1.82 (1.70-1.94)	0.72	0.2996
Acceptance	2.00	1.86 (1.79-1.93)	0.66	2.00	1.88 (1.77-2.00)	0.66	0.9783
Humour	1.00	0.96 (0.88-1.03)	0.69	0.50	0.82 (0.70-0.94)	0.70	0.0232*
Religion	1.50	1.36 (1.26-1.47)	0.95	1.00	1.09 (0.92-1.27)	1.01	0.0043**
Emotional support	2.00	1.90 (1.82-1.98)	0.72	2.00	1.89 (1.74-2.05)	0.88	0.6478
Instrumental supports	2.00	1.87 (1.78-1.95)	0.75	2.00	1.65 (1.49-1.81)	0.95	0.0531
Self-distraction	2.00	1.79 (1.72-1.86)	0.66	2.00	1.74 (1.62-1.87)	0.74	0.9602
Denial	1.00	0.98 (0.91-1.06)	0.71	0.50	0.66 (0.56-0.77)	0.60	0.0000***
Venting	1.50	1.36 (1.29-1.43)	0.62	1.00	1.20 (1.08-1.32)	0.71	0.0358*
Substance use	0.00	0.42 (0.34-0.50)	0.70	0.00	0.44 (0.30-0.57)	0.77	0.8739
Behavioural disengagement	0.50	0.78 (0.71-0.85)	0.65	1.00	0.79 (0.67-0.91)	0.69	0.8923
Self-blame	1.00	1.15 (1.06-1.23)	0.82	1.00	1.13 (1.00-1.26)	0.73	0.8980

p - test probability value * p<0.05, ** p<0.01, *** p<0.001.

The use of stress-management strategies with the fact of working in a medical facility where refugees stayed is presented in **Table 11**. Again, the use of planning strategies was slightly more frequent, while denial and religion were less often applied.

**Table 11:** Coping strategies among nurses working in a medical facility where war refugees stayed.

Mini-COPE	Working in a medical facility where war refugees stayed						*p*
	no (*N* = 314)			yes (*N* = 151)			
	Median	Mean (95% c.i.)	Std. dev.	Median	Mean (95% c.i.)	Std. dev.	
Active coping	2.00	2.10 (2.03-2.16)	0.58	2.00	2.08 (1.98-2.19)	0.66	0.8764
Planning	2.00	2.01 (1.94-2.08)	0.67	2.00	2.15 (2.05-2.26)	0.66	0.0415*
Positive reframing	2.00	1.75 (1.67-1.83)	0.69	2.00	1.80 (1.69-1.91)	0.70	0.4722
Acceptance	2.00	1.87 (1.79-1.94)	0.65	2.00	1.86 (1.76-1.97)	0.67	0.7884
Humour	1.00	0.94 (0.87-1.02)	0.68	0.50	0.88 (0.77-1.00)	0.72	0.1816
Religion	1.50	1.37 (1.27-1.48)	0.95	1.00	1.09 (0.93-1.25)	0.99	0.0018**
Emotional support	2.00	1.90 (1.82-1.98)	0.72	2.00	1.92 (1.78-2.06)	0.86	0.3804
Instrumental supports	2.00	1.87 (1.78-1.95)	0.75	2.00	1.68 (1.53-1.83)	0.93	0.0725
Self-distraction	2.00	1.80 (1.73-1.87)	0.64	2.00	1.75 (1.62-1.87)	0.77	0.9997
Denial	1.00	0.96 (0.88-1.03)	0.70	0.50	0.77 (0.66-0.88)	0.67	0.0057**
Venting	1.50	1.35 (1.28-1.42)	0.62	1.50	1.25 (1.14-1.36)	0.69	0.2086
Substance use	0.00	0.40 (0.32-0.47)	0.68	0.00	0.48 (0.36-0.61)	0.79	0.3306
Behavioural disengagement	0.50	0.78 (0.71-0.85)	0.63	1.00	0.81 (0.70-0.93)	0.74	0.8605
Self-blame	1.00	1.14 (1.05-1.23)	0.81	1.00	1.12 (1.00-1.24)	0.75	0.9502

p - test probability value * p<0.05, ** p<0.01.

There was no statistically significant relationship between taking up additional work with refugees and stress-management strategies according to Mini-COPE (**Table 12**).

**Table 12:** Coping strategies among nurses taking up additional work to help refugees.

Mini-COPE	Taking up additional work to help refugees						*p*
	no (*N* = 411)			yes (*N* = 50)			
	Median	Mean (95% c.i.)	Std. dev.	Median	Mean (95% c.i.)	Std. dev.	
Active coping	2.00	2.09 (2.03-2.15)	0.60	2.00	2.16 (1.97-2.35)	0.67	0.4798
Planning	2.00	2.06 (2.00-2.13)	0.67	2.00	2.08 (1.89-2.27)	0.66	0.9053
Positive reframing	2.00	1.76 (1.69-1.83)	0.71	2.00	1.84 (1.67-2.01)	0.59	0.3917
Acceptance	2.00	1.88 (1.82-1.95)	0.66	2.00	1.87 (1.71-2.03)	0.58	0.5024
Humour	1.00	0.92 (0.85-0.98)	0.69	1.00	0.90 (0.70-1.10)	0.71	0.7673
Religion	1.00	1.26 (1.16-1.35)	0.96	1.50	1.45 (1.16-1.74)	1.03	0.1988
Emotional support	2.00	1.90 (1.82-1.97)	0.77	2.00	1.98 (1.77-2.19)	0.73	0.3221
Instrumental supports	2.00	1.79 (1.71-1.87)	0.82	2.00	1.94 (1.73-2.15)	0.75	0.2158
Self-distraction	2.00	1.78 (1.72-1.85)	0.67	2.00	1.79 (1.57-2.01)	0.78	0.6709
Denial	1.00	0.88 (0.82-0.95)	0.70	1.00	0.97 (0.80-1.14)	0.60	0.2714
Venting	1.50	1.32 (1.26-1.39)	0.65	1.50	1.32 (1.14-1.50)	0.62	0.8765
Substance use	0.00	0.41 (0.34-0.47)	0.71	0.00	0.53 (0.32-0.74)	0.75	0.1491
Behavioural disengagement	0.50	0.77 (0.71-0.84)	0.66	0.75	0.74 (0.56-0.92)	0.62	0.8324
Self-blame	1.00	1.14 (1.07-1.22)	0.79	1.00	1.00 (0.79-1.21)	0.76	0.2714

## 4 Discussion

The aim of this study was to examine the stress levels experienced by Polish nurses working with Ukrainian war refugees and to analyze how their involvement influenced their use of coping strategies. Specifically, the study sought to identify whether contact with refugees was associated with increased stress and to explore the coping mechanisms adopted by nurses in such circumstances.

The average stress level among nurses was moderate, with nearly equal proportions of nurses experiencing low, medium, and high stress levels. A study by Adriaenssens, De Gucht, and Maes (15) found that emergency nurses often experience high levels of stress due to the nature of their work, with stress scores frequently falling in the moderate to high range. This aligns with the current study, where a substantial proportion of nurses reported moderate to high stress levels. A study by Shirey (16) in the United States reported that nurses frequently experience moderate to high stress levels due to job demands, work environment, and emotional labor. Van der Heijden et al. (17) found similar results, indicating that nurses across different countries in Europe experience comparable levels of stress due to workload, patient care demands, and organizational factors. The findings of moderate to high stress levels in this studies are consistent with the current research.

The study indicated that nurses who had contact with refugees in the last six months and/or helped them, did not present an increased level of stress - on the contrary, this level was lower. This finding is intriguing and somewhat counterintuitive. Typically, dealing with traumatic situations or vulnerable populations, such as refugees, might be expected to increase stress levels. The reasons for this seemingly paradoxical relationship can be explained firstly by the fact that where there was freedom of choice, individuals with lower stress levels were the first to start working with war refugees. This aligns with theories on altruism and helping behavior, where assisting others, particularly those in distress, has been shown to foster a sense of personal achievement and lower stress levels (18),. Secondly, working with refugees and having direct contact with them probably had a calming effect – just as people from the western part of Poland showed greater symptoms of panic due to the war in Ukraine than those living in the Subcarpathian region directly bordering with Ukraine (19).

A study by Lusk and Fater (20) found that nurses working with trauma patients, including refugees, often reported higher stress and burnout levels due to the emotional and psychological challenges. A systematic review by Kavukcu and Altıntaş (21) show that healthcare workers working with war refugees reported increased stress due to the emotional burden and the complexity of care required. This contrasts with the current study’s finding that such contact reduced stress levels, suggesting that the specific context and support systems available to nurses play a crucial role. In contrast, Apostolara et al. (22) and Mavratza et al. (4) reported that healthcare workers involved in refugee care in Greece experienced lower stress levels when they perceived their work as meaningful and received adequate support from their institutions, aligning with the current study’s findings.

The study’s regression analysis identified age and education level as significant factors in reducing stress levels. Nurses with a master’s degree and older nurses reported lower stress levels. This finding is supported by several studies, including one by Jennings (23), which found that experienced nurses and those with higher education levels often have better coping mechanisms and lower stress levels. Research by McVicar (24) supports this, showing that experienced nurses and those with advanced qualifications tend to have better stress management strategies and lower stress levels. This is due to increased competence, confidence, and coping mechanisms developed over time. Similarly, a study by Jachens, Houdmont and Thomas (25) found that higher education levels correlate with better job satisfaction and lower stress, as advanced education equips nurses with improved skills and knowledge for handling stressors effectively.

The over 10% greater share of low-stress levels among nurses helping refugees suggests a potential stress-buffering effect of such work. The review by Dodds & Hunter (26) showed that in similar studies, researchers obtained opposite results.

The data from 149 nurses who worked with refugees in a medical facility provides valuable insights into whether perceiving the work with refugees as a burden influences their stress levels, as measured by the Perceived Stress Scale (PSS-10). There were 97 nurses who did not feel that working with refugees was a burden and 48 nurses who felt that working with refugees was a burden. Interestingly, the mean stress level is slightly lower among nurses who felt that working with refugees was a burden (15.0) compared to those who did not feel burdened (16.9). This result is counterintuitive, as one might expect those feeling burdened to report higher stress levels.

Nurses who perceive their work as a burden might have developed coping mechanisms that mitigate stress, leading to lower or comparable stress levels despite feeling burdened. Nurses who do not feel burdened may be more intrinsically motivated and possibly more invested in their work, which can sometimes lead to higher stress due to higher expectations and emotional involvement. The subjective perception of burden might not always align with actual stress levels. Factors such as personal resilience, support systems, and individual differences in stress perception and management can play significant roles.

The findings align with several studies in the field. Kiselev et al. (27) found that healthcare workers involved in refugee care experienced lower stress levels when they perceived their work as meaningful and received adequate support from their institutions. Irfan et al. (28) and Yang et al. (29) reported that proactive coping strategies and meaningful engagement in work during the COVID-19 pandemic significantly reduced stress among healthcare workers. Labrague et al. (30) highlighted the importance of emotional and instrumental support in reducing stress among nurse managers, suggesting that the perception of burden might be mitigated by effective support systems.

Overall, the findings suggest that interaction with war refugees does not increase stress and may slightly reduce it. Factors such as education level and age play a more significant role in determining stress levels among nurses.

The results of our study show that the frequency of using active (constructive) strategies is much higher than avoidant (destructive) ones – for example, denial, substance use, or passive strategies – self-blame or behavioral disengagement. The research conducted among nurses across various countries yielded several notable conclusions. Consistent with our study, active coping strategies were reported by Tesfaye (31), Siemianowska et al. (32), Chang et al. (33). Conversely, avoidant strategies were least frequently employed in the research by Beh (34), Tesfaye (35), Siemianowska et al. (32) and Bjorvatan et al. (35), aligning with our findings. Divergent results were observed in studies conducted by Jan (36) and Haslinda et al. (37), where support-seeking and emotion-focused strategies were more prevalent. Additionally, research conducted in Chang et al. (38) indicated a higher use of avoidant coping strategies among nurses.

The relationship between the use of stress-management strategies at work with refugees and the fact of providing help to refugees was examined. Three questions regarding assistance and contacts with refugees were taken into consideration. Results shows that the nurses who helped refugees at work more often used a planning strategy in stressful situations, and less frequently reacted to stress using humour, religion, denial or venting. There were no statistically significant relationships for the remaining strategies. Similar results received when comparing the use of stress-management strategies with the fact of working in a medical facility where refugees stayed. Again, the use of planning strategies was slightly more frequent, while denial and religion were less often applied. Planning strategy according to research conducted by Vukčević Marković and Živanović (39) positively affects the mental health of nurses working with refugees.

The findings of this study can be discussed through the lens of the Demand, Control, and Social Support Model (40). Polish nurses faced high job demands, especially in terms of the increased workload and emotional strain of caring for Ukrainian war refugees. The results highlight that although these nurses experienced work fatigue, their stress levels were paradoxically lower when they were more actively involved in refugee care. This can be explained by the control element of the model: nurses who had direct contact with refugees likely felt a greater sense of autonomy and control over their work. By actively engaging in meaningful tasks, such as planning patient care and solving complex problems, they had more opportunities to exercise decision-making, which may have contributed to their reduced stress levels. The social support component of the model also plays an important role in this dynamic. The sense of fulfillment derived from helping refugees and the perception of performing a meaningful duty acted as psychological support, buffering against the negative effects of high job demands. This aligns with Karasek’s model, which posits that high levels of social support—whether from colleagues, supervisors, or the nature of the work itself—can mitigate stress caused by demanding job conditions. In conclusion, the study’s results are consistent with the Demand, Control, and Social Support Model, demonstrating that while job demands were high, the combination of control over their work and the social and emotional support gained from meaningful engagement helped nurses manage stress effectively.

There was no statistically significant relationship between taking up additional work with refugees and stress-management strategies. Perhaps the lack of this significance is due to the relatively small number of nurses who answered affirmatively to the question about taking up additional work.

After discussion of the obtained results, a reflection comes to mind that contacts with refugees could have changed the approach to stressful situations. The relationship could have been in the opposite direction - people with a certain approach to stressful situations could have been more involved in helping refugees.

Collaboration between healthcare providers and the host community is crucial in promoting the well-being of refugees. Actions such as ensuring access to healthcare, offering psychological support, and facilitating social integration should be integral to both health policies and nurse training programs (41).

The results of this study have significant implications for both health policy and nurse education programs. The findings emphasize the necessity for implementing policies that focus on stress management and coping strategies for nurses, particularly those working with vulnerable populations, such as war refugees. It is essential for health systems to invest in mental health support and resilience training for healthcare professionals, as direct involvement with refugees has been shown to potentially lower stress levels when appropriate coping mechanisms are in place. Therefore, policies should encourage the use of active coping strategies, such as planning and problem-solving.

In the context of nursing education, the study highlights the importance of integrating psychosocial care and trauma-informed approaches into the curriculum. Training programs should focus on fostering resilience, cultural competence, and strategies to maintain well-being in high-stress environments. Practical components, including case studies, simulations, and hands-on exercises related to refugee care, are essential to prepare nurses for such challenges.

## 5 Methodological limitations

Methodological limitations of this study include the sample size, the cross-sectional design, which prevents the identification of causal relationships between variables, and the reliance on self-report questionnaires. The voluntary nature of participation introduces potential bias, and the reasons for non-participation by 21.2% of the invited nurses remain unknown, which could further affect the representativeness of the sample.

The validation of the results of this study, could be performed with longitudinal studies and comparison with control groups. Conducting follow-up assessments over time could validate the stability of the results, particularly the relationship between contact with refugees and stress levels. A longitudinal approach would help determine whether the reduction in stress persists or fluctuates as job demands change. Including a control group of nurses from other regions could help confirming the results’ significance.

To reduce the impact of social desirability bias, which may cause participants to give answers that reflect positively on themselves (e.g., reporting lower stress or more active coping) indirect questioning techniques can encourage more honest responses.

The results can be cautiously generalized to nurses in other regions or countries facing similar crises, but with certain limitations. Since the study was conducted in only one region of Poland, the findings may not fully represent the experiences of nurses in other parts of the country or in different healthcare systems. Factors like regional healthcare infrastructure, local support systems, and cultural attitudes towards refugee care may influence outcomes. Expanding the study to multiple regions or countries with diverse healthcare systems would provide more robust and generalizable results.

## 6 Conclusions

6.1. Polish nurses who had contact with or assisted Ukrainian war refugees reported lower levels of stress compared to those who did not engage with refugees.
6.2. Although the increased workload and frequent contact with Ukrainian refugees contributed to work-related fatigue, paradoxically, it also reduced stress by providing a sense of fulfillment from performing a meaningful duty.
6.3. Nurses who assisted Ukrainian war refugees more often employed active coping strategies, such as planning and problem-solving, and were less likely to resort to avoidant strategies.
6.4. Empowering nurses through greater autonomy and fostering supportive work environments, especially in crisis situations like the care of war refugees has a positive impact on coping with stress.

## Data Availability

The raw data supporting the conclusions of this article will be made available by the authors, without undue reservation.
